# M2 macrophages, but not M1 macrophages, support megakaryopoiesis by upregulating PI3K-AKT pathway activity

**DOI:** 10.1038/s41392-021-00627-y

**Published:** 2021-06-18

**Authors:** Hong-Yan Zhao, Yuan-Yuan Zhang, Tong Xing, Shu-Qian Tang, Qi Wen, Zhong-Shi Lyu, Meng Lv, Yu Wang, Lan-Ping Xu, Xiao-Hui Zhang, Yuan Kong, Xiao-Jun Huang

**Affiliations:** 1 Peking University People’s Hospital, Peking University Institute of Hematology, National Clinical Research Center for Hematologic Disease, Beijing Key Laboratory of Hematopoietic Stem Cell Transplantation, Collaborative Innovation Center of Hematology, Peking University, Beijing, China; 2 Peking-Tsinghua Center for Life Sciences, Academy for Advanced Interdisciplinary Studies, Peking University, Beijing, China

**Keywords:** Translational research, Haematological diseases

## Abstract

Dysfunctional megakaryopoiesis hampers platelet production, which is closely associated with thrombocytopenia (PT). Macrophages (MФs) are crucial cellular components in the bone marrow (BM) microenvironment. However, the specific effects of M1 MФs or M2 MФs on regulating megakaryocytes (MKs) are largely unknown. In the current study, aberrant BM-M1/M2 MФ polarization, characterized by increased M1 MФs and decreased M2 MФs and accompanied by impaired megakaryopoiesis-supporting abilities, was found in patients with PT post-allotransplant. RNA-seq and western blot analysis showed that the PI3K-AKT pathway was downregulated in the BM MФs of PT patients. Moreover, in vitro treatment with PI3K-AKT activators restored the impaired megakaryopoiesis-supporting ability of MФs from PT patients. Furthermore, we found M1 MФs suppress, whereas M2 MФs support MK maturation and platelet formation in humans. Chemical inhibition of PI3K-AKT pathway reduced megakaryopoiesis-supporting ability of M2 MФs, as indicated by decreased MK count, colony-forming unit number, high-ploidy distribution, and platelet count. Importantly, genetic knockdown of the PI3K-AKT pathway impaired the megakaryopoiesis-supporting ability of MФs both in vitro and in a MФ-specific PI3K-knockdown murine model, indicating a critical role of PI3K-AKT pathway in regulating the megakaryopoiesis-supporting ability of M2 MФs. Furthermore, our preliminary data indicated that TGF-β released by M2 MФs may facilitate megakaryopoiesis through upregulation of the JAK2/STAT5 and MAPK/ERK pathways in MKs. Taken together, our data reveal that M1 and M2 MФs have opposing effects on MKs in a PI3K-AKT pathway-dependent manner, which may lead to new insights into the pathogenesis of thrombocytopenia and provide a potential therapeutic strategy to promote megakaryopoiesis.

## Introduction

Megakaryopoiesis is the process by which hematopoietic stem cells (HSCs) migrate from the osteoblastic microenvironment to the vascular microenvironment and eventually differentiate into megakaryocytes (MKs), which then undergo proliferation, differentiation, and maturation to produce platelets in bone marrow (BM).^1–3^ Emerging evidence from murine studies suggests that effective megakaryopoiesis depends on a harmonious BM microenvironment.^3–5^ By contrast, platelet production is impeded by dysfunctional megakaryopoiesis, which is closely associated with poor MK engraftment after allogeneic hematopoietic stem cell transplantation (allo-HSCT).^6–8^ We recently reported that an impaired BM microenvironment is responsible for the occurrence of prolonged isolated thrombocytopenia (PT), which is characterized by dysfunctional MK maturation and thrombocytopenia.^7–10^ Moreover, in vitro and in vivo treatments for restoring the impaired BM microenvironment promoted MK maturation in PT patients,^8–10^ further confirming the vital role of the BM microenvironment in supporting megakaryopoiesis.

Macrophages (MΦs) are crucial cellular components that support hematopoiesis in the BM microenvironment.^11–14^ However, BM MΦs were reported to play both positive and negative roles in regulating megakaryopoiesis.^15–17^ In patients with immune thrombocytopenia (ITP) who underwent intravenous immunoglobulin therapy, BM MΦs were found to closely contact MKs and were associated with increased platelet counts, suggesting that MΦs positively regulate megakaryopoiesis.^15^ However, Alves-Rosa et al. reported that depleting BM MΦs enhanced both megakaryopoiesis and platelet production in an ITP mouse model, which indicates that MΦs negatively regulate megakaryopoiesis.^16,17^ Typically, MΦs can be polarized into classically activated (M1) MΦs and alternatively activated (M2) MΦs, with distinct phenotypic and unique functional characteristics.^18^ M1 MΦs mediate resistance to intracellular pathogens and tissue destruction, whereas M2 MΦs are generally oriented to tissue remodeling and repair.^19,20^ Recently, a confocal laser scanning microscopy study revealed an increased number of M1 MΦs and a decreased number of M2 MΦs in the spleens of ITP patients compared with non-ITP control patients, providing clues that M1 MΦs and M2 MΦs have different roles in regulating thrombopoiesis.^21^ However, the specific effects of M1 or M2 MΦs on regulating megakaryopoiesis and approaches for regulating the supportive function of MΦs in megakaryopoiesis need to be further elucidated.

The PI3K-AKT pathway is a central signaling pathway for cellular growth and survival. Animal studies using knockout mice or small interfering RNAs demonstrated that the PI3K-AKT pathway is involved in the regulation of M1/M2 MФ polarization, especially with regard to the activation of M2 MФs.^22,23^ Furthermore, considerable evidence has implicated the PI3K-AKT pathway in the survival, proliferation, and metabolism of M2 MФs. Munugalavadla et al.^24^ reported that MФs from PI3K-deficient mice exhibit significantly reduced growth and migration abilities. Chang et al.^25^ found that BM MФs maintain their survival by upregulating glucose uptake, which is dependent on the PI3K-AKT pathway. Although accumulating evidence indicates that the PI3K-AKT pathway plays a crucial role in promoting M2 polarization to reprogram MФ function, the effect of the PI3K-AKT pathway on regulating the megakaryopoiesis-supporting ability of MФs has not been studied in MФ subtypes.

Therefore, the current study was performed to address the roles of M1 MФs and M2 MФs in regulating megakaryopoiesis. The effect of the PI3K-AKT pathway on regulating the megakaryopoiesis-supporting ability of MФs was investigated in vitro and in a MΦ-specific PI3K-knockdown murine model. Moreover, the role of M1/M2 MФ polarization in regulating megakaryopoiesis and pharmacological interventions of the PI3K-AKT pathway were evaluated in PT patients, a clinical model of thrombocytopenia after allo-HSCT. We aimed to identify a potential therapeutic strategy for patients with dysfunctional MK maturation and thrombocytopenia.

## Results

### Altered distribution of monocyte-MΦ subsets, especially increased M1 MΦs and decreased M2 MΦs in the BM of PT patients

**Fig. 1:**
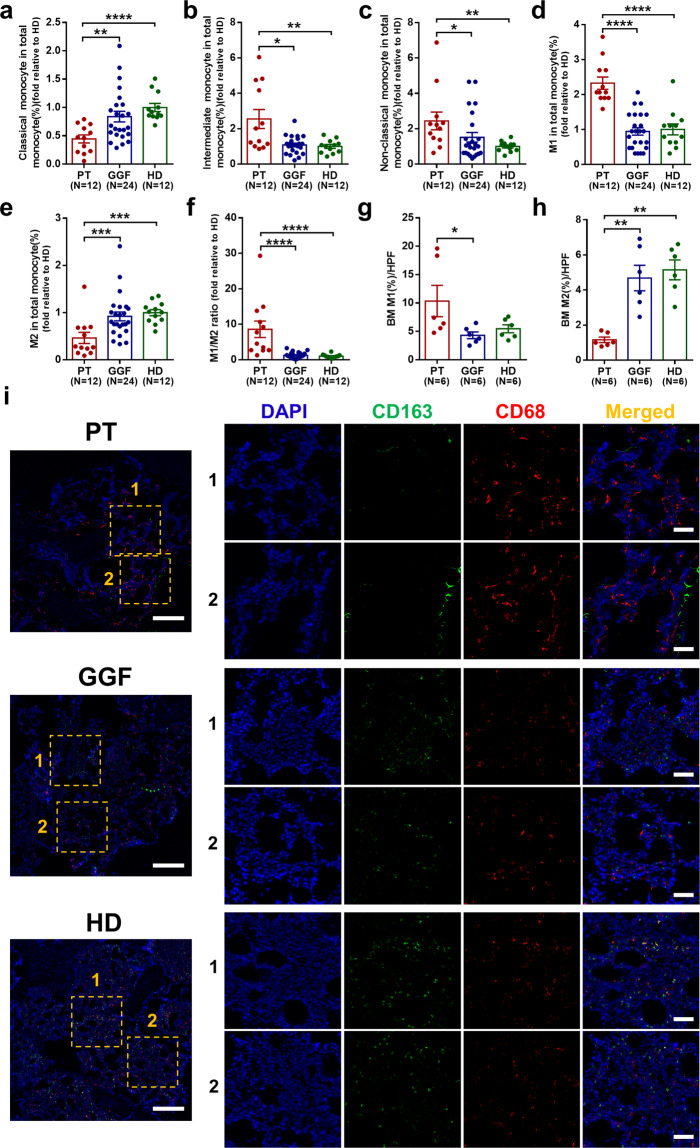
Altered distribution of monocyte–macrophage subsets in the bone marrow of PT patients. Percentage of **a** classical monocytes, **b** intermediate monocytes, **c** non-classical monocytes, **d** M1 MФs, and **e** M2 MФs in the BM of PT patients, GGF patients and HDs as indicated by flow cytometry. **f** The M1/M2 ratio was calculated to represent the MФ polarization status in PT patients, GGF patients, and HD. Positive area of **g** CD68^+^ M1 MФs and **h** CD163^+^ M2 MФs per high-power field[HPF] in the BM trephine biopsies of PT patients, GGF patients, and HD as quantified by ImageJ. **i** M1 (CD68^+^, red) and M2 (CD163^+^, green) MФs in BM trephine biopsies from PT patients, GGF patients, and HD were visualized by immunofluorescence. Nuclei were counterstained with DAPI (blue). An overlay of the three colors reveals the number of M1 and M2 MФs in the BM trephine biopsies from the three groups (scale bars represent 50 μm). The images (right panel) are enlarged from the labeled square areas of the left images. Data are presented as the means ± SEM (*P ≤ 0.05, ** *P* ≤ 0.01, *** *P* ≤ 0.001, **** *P* ≤ 0.0001)

To investigate the functional role of MΦs in patients with PT, a clinical manifestation of megakaryopoiesis failure after allo-HSCT, representative monocyte-MΦ subsets were analyzed by flow cytometry (Supplementary Fig. S1a–c). Decreased percentages of classical monocytes (Fig. 1a; 0.45 ± 0.07-fold vs. 0.84 ± 0.09-fold, *P* = 0.008), increased percentages of intermediate monocytes (Fig. 1b; 2.55 ± 0.54-fold vs. 1.08 ± 0.10-fold, *P* = 0.02) and non-classical monocytes (Fig. 1c; 2.44 ± 0.50-fold vs. 1.52 ± 0.27-fold, *P* = 0.03) were observed in BM of PT patients compared with good graft function (GGF) patients. Aberrant M1/M2 MФ polarization of pre-cultivated BM MΦs, characterized by a significant increase in the number of M1 MΦs (Fig. 1d; 2.33 ± 0.18-fold vs. 0.95 ± 0.10-fold, *P* < 0.0001) and a decrease in the number of M2 MΦs (Fig. 1e; 0.46 ± 0.12-fold vs. 0.92 ± 0.09-fold, *P* = 0.0004), was observed in PT patients compared with GGF patients. Therefore, the M1/M2 ratio was markedly higher in PT patients than in GGF patients (Fig. 1f; 8.60 ± 2.31-fold vs. 1.27 ± 0.18-fold, *P* < 0.0001).

In situ histological analyses of the BM trephine biopsies (BMBs) were performed to further characterize the pre-cultivated BM MΦs. Consistent with our flow cytometry data, immunofluorescence staining revealed that the percentage of CD68^+^ M1 MΦs per high-power field (HPF) in BMBs increased significantly (Fig. 1g, i; 10.35% ± 2.76% vs. 4.31% ± 0.60%, *P* = 0.03), whereas that of CD163^+^ M2 MΦs reduced significantly (Fig. 1h, i; 1.16% ± 0.16% vs. 4.69% ± 0.73%, *P* = 0.002) in PT patients compared with GGF patients. Taken together, these results demonstrated a significant increase in the M1/M2 MΦ ratio, which is driven by the increased M1 MФs and decreased M2 MФs in the BM microenvironment of PT patients.

### Increased M1 MФs and decreased M2 MФs lead to a reduced megakaryopoiesis-supporting ability in the BM of PT patients

**Fig. 2:**
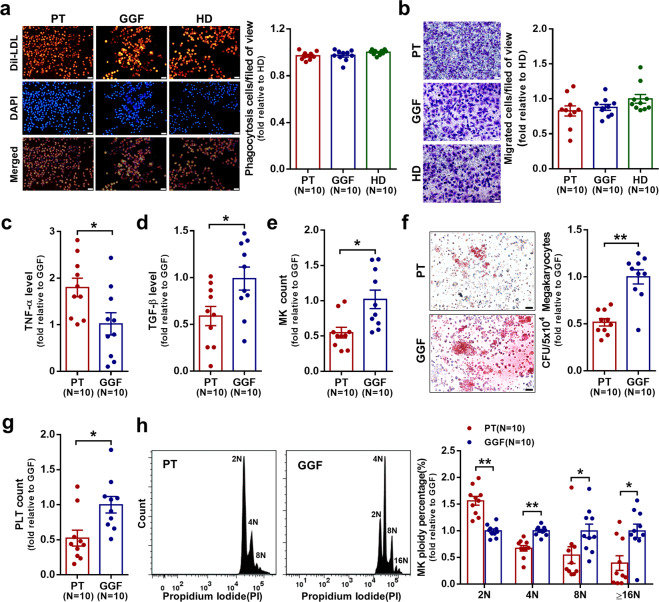
Increased M1 macrophages and decreased M2 macrophages lead to a reduced megakaryopoiesis-supporting ability in the bone marrow of PT patients. **a** Representative images (left panel, scale bars represent 50 μm) and quantification (right panel) of the phagocytosis assays for the cultivated BM MФs from PT patients, GGF patients and HD after 7 days in culture. **b** Representative images (left panel, scale bars represent 50 μm) and quantification (right panel) of the transwell migration assays for the cultivated BM MФs from PT patients, GGF patients, and HD after 7 days in culture. The intracellular levels of **c** TNF-ɑ and **d** TGF-β were analyzed in BM MФs from PT patients and GGF patients after 7 days of culture. MK production and maturation, colony-forming unit MK (CFU-MK) plating efficiencies, and platelet release were analyzed after coculture with the cultivated BM MФs from PT patients and GGF patients. The **e** MK count, **f** CFU-MK count (left panel: representative images, scale bars represent 50 μm; right panel: quantification), **g** platelet count and **h** MK ploidy distribution were analyzed after 12 days of coculture. Data are presented as the means ± SEM (*P ≤ 0.05, **P ≤ 0.01)

As shown in Supplementary Fig. S1d, the phenotypes of MФs cultured from monocytes isolated from the BM (cultivated BM MФs) of PT patients (PT MФs) were dominated by M1 MФs (CD68^+^CCR2^+^), whereas the cultivated GGF MФs were dominant by M2 MФs (CD163^+^CX3CR1^+^). The phagocytic (Fig. 2a) and migratory (Fig. 2b) functions of the cultivated BM MФs from PT patients were not significantly different from those from GGF patients and healthy donors (HD). The level of intracellular TNF-ɑ (Fig. 2c; 1.80 ± 0.20-fold, *P* = 0.02) was significantly higher and that of TGF-β (Fig. 2d; 0.58 ± 0.10-fold, *P* = 0.04) was remarkably lower in PT MФs than in GGF MФs.

To explore whether the distribution of MФ subsets affects megakaryopoiesis in PT patients, coculture experiments of the MKs, which were differentiated from BM CD34^+^ cells of HD with the cultivated BM MФs of PT patients or GGF patients were performed. Moreover, the coculture of BM CD34^+^ cells with PT MФs showed significant decreases in the MK count (Fig. 2e; 0.55 ± 0.08-fold, *P* = 0.01), number of CFU-MK (Fig. 2f; 0.52 ± 0.04-fold, *P* = 0.007) and platelet count (Fig. 2g; 0.52 ± 0.11-fold, *P* = 0.03) compared with those cocultured with GGF MФs. MKs cocultured with PT MФs had significantly more 2N MKs (Fig. 2h; 1.56 ± 0.08-fold, *P* = 0.005) and significantly fewer 4N MKs (Fig. 2h; 0.67 ± 0.05-fold, *P* = 0.005), 8N MKs (Fig. 2h; 0.55 ± 0.16-fold, *P* = 0.03), and ≥16N MKs (Fig. 2h; 0.40 ± 0.14-fold, *P* = 0.02) than those MKs cocultured with GGF MФs. This finding indicates that aberrant BM-M1/M2 MФ polarization, characterized by increased M1 MФs and decreased M2 MФs may hamper megakaryopoiesis in the BM microenvironment of PT patients.

### Downregulation of the PI3K-AKT pathway is involved in the aberrant BM-M1/M2 MФ polarization of PT patients

**Fig. 3:**
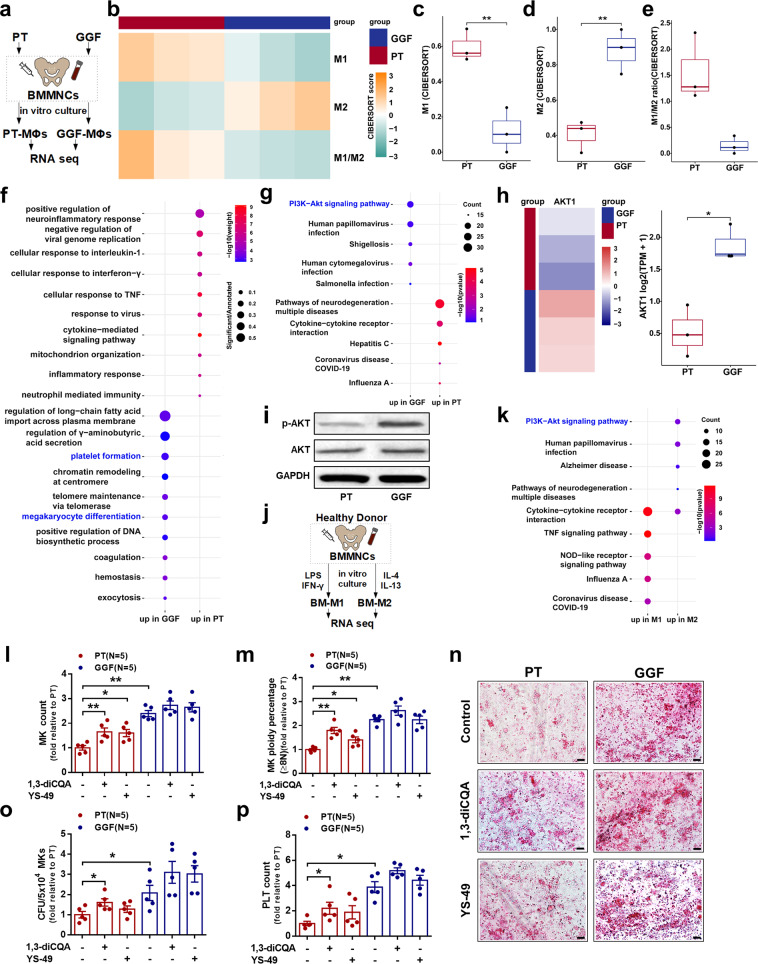
Activating the PI3K-AKT pathway enhanced the megakaryopoiesis-supporting ability of macrophages from PT patients. **a** Schematic diagram of the study design on RNA-seq of the cultivated BM MФs from PT and GGF patients. **b** Heatmaps showed M1 and M2 scores (calculated by CIBERSORT algorithm) in PT MФs and GGF MФs (scaled by row). Box plot depicting **c** CIBERSORT score of M1 MФs, **d** CIBERSORT score of M2 MФs and **e** CIBERSORT score of M1/M2 in PT MФs and GGF MФs. **f** GO enrichment analysis showed the top ten terms enriched by the upregulated genes of GGF MФs and PT MФs. The size of each circle indicates the ratio of DEGs counts and the gene counts of the term. **g** KEGG enrichment analysis showed the top five pathways enriched by the upregulated genes of GGF MФs and PT MФs. **h** Heatmap showed expression of AKT1 gene in GGF MФs and PT MФs. **i** Representative western blots of p-AKT, AKT, and GAPDH expression in the cultivated BM MФs from PT patients and GGF patients. **j** Schematic diagram of the study design on RNA-seq of BM-M1 and BM-M2 from HD. **k** KEGG enrichment analysis showed the top five pathways enriched by the upregulated genes of M1 MФs and M2 MФs. The size of each circle indicates the scaled ratio of DEGs counts and the gene counts of the pathway. MK production and maturation, CFU-MK plating efficiencies, and platelet release were analyzed after coculture with BM MФs that were cultivated from PT patients and GGF patients and were subjected to various treatments. The **l** MK count, **m** MK ploidy distribution, **n** representative CFU-MK images (scale bars represent 50 μm), **o** CFU-MK count, and **p** platelet count were analyzed after 12 days of coculture. Data are presented as the means ± SEM (*P ≤ 0.05, ** *P* ≤ 0.01)

To verify the MФ polarization status with another more comprehensive method, the M1 and M2 score, which represents M1 and M2 phenotype, was estimated by bulk RNA sequencing (RNA-seq) data of the cultivated BM MФs (the calculation details and concept definitions are provided in the “Materials and methods” section). Consistent with our flow cytometry data, RNA-seq revealed that PT MФs were dominated by M1 MФs, whereas GGF MФs were dominant by M2 MФs (Fig. 3a, b). M1 score was significantly higher in PT MФs than that in GGF MФs (Fig. 3c; 0.60 ± 0.05 vs. 0.12 ± 0.07, *P* = 0.008). Conversely, the M2 score was significantly higher in GGF MФs than that in PT MФs (Fig. 3d; 0.88 ± 0.07 vs. 0.40 ± 0.05, *P* = 0.008). Thus, the ratio of M1 to M2 score was higher in PT MФs than that in GGF MФs (Fig. 3e; 1.57 ± 0.38 vs. 0.15 ± 0.10, *P* = 0.056). RNA-seq data supported that PT MФs were imbalanced polarized to M1 MФ type. Gene ontology (GO) analysis indicated that the upregulated differentially expressed genes in GGF MФs are particularly enriched in megakaryopoiesis-associated biological processes (Fig. 3f). Next, the Kyoto Encyclopedia of Genes and Genomes (KEGG) pathway gene set data were analyzed. Compared with PT MФs, GGF MФs showed significant upregulation of the PI3K-AKT pathway (Fig. 3g). Moreover, AKT1 was highly expressed in GGF MФs than in PT MФs (Fig. 3h; 1.87 ± 0.28 vs. 0.52 ± 0.40, *P* = 0.01). Consistent with RNA-seq results, a lower level of p-AKT was observed in PT MФs compared to that in GGF MФs by western blot analysis (Fig. 3i).To further confirm whether the PI3K-AKT pathway potentially upregulated in M2 MФs, M1 MФs, and M2 MФs from HD BM were investigated using RNA-seq (Fig. 3j). As shown in Fig. 3k, KEGG enrichment analysis showed that upregulated genes were mainly enriched in PI3K-AKT pathway in the M2 MФs. Taken together, these data indicate that the PI3K-AKT pathway may be a target to improve the megakaryopoiesis-supporting ability of PT MФs.

### Activating the PI3K-AKT pathway enhanced the megakaryopoiesis-supporting ability of BM MФs from PT patients

To investigate whether activating the PI3K-AKT pathway enhances the megakaryopoiesis-supporting ability of BM MФs from PT patients, BM CD34^+^ cells from HD were cocultured with PT MФs treated with 1,3-dicaffeoylquinic acid (1,3-diCQA) or YS-49 (PI3K-AKT pathway activators). Both 1,3-diCQA and YS-49 improved the ability of BM MФs from PT patients to support megakaryopoiesis, as indicated by increases in MK count (Fig. 3l; 1.65 ± 0.16-fold, *P* = 0.008; 1.6 ± 0.15-fold, *P* = 0.02), percentage of high-ploidy MKs (≥8N) (Fig. 3m; 1.80 ± 0.13-fold, *P* = 0.008; 1.40 ± 0.12-fold, *P* = 0.02), number of CFU-MK (Fig. 3n, o; 1.61 ± 0.19-fold, *P* = 0.05; 1.28 ± 0.16-fold, *P* = 0.22), and platelet count (Fig. 3p; 2.20 ± 0.48-fold, *P* = 0.02; 1.89 ± 0.52-fold, *P* = 0.22). These results suggest that activating the PI3K-AKT pathway improves the function of BM MФs from PT patients, with a particular increase in their megakaryopoiesis-supporting ability.

### BM-M1 and BM-M2 exerted opposing effects on megakaryopoiesis and platelet production in vitro

To investigate the effects of M1 MФs and M2 MФs on megakaryopoiesis, primary BM MФs (BM-M0) from HD were polarized into M1 MФs (BM-M1) or M2 MФs (BM-M2). Then, both direct- and indirect-contact coculture experiments of the MKs, which were differentiated from BM CD34^+^ cells of HD, with BM-M0, BM-M1 or BM-M2 were performed, respectively. As shown in Supplementary Fig. S2a, the phenotypes of M1 MФs (CD68^+^CCR2^+^) and M2 MФs (CD163^+^CX3CR1^+^) were confirmed by flow cytometry. The levels of intracellular TNF-ɑ (Supplementary Fig. S2b; 0.44 ± 0.04-fold, *P* = 0.002) and IL-12 (Supplementary Fig. S2c; 0.51 ± 0.06-fold, *P* = 0.03) were significantly lower, whereas TGF-β (Supplementary Fig. S2d; 3.01 ± 0.12-fold, *P* = 0.002) was remarkably higher in BM-M2 than in BM-M1.

**Fig. 4:**
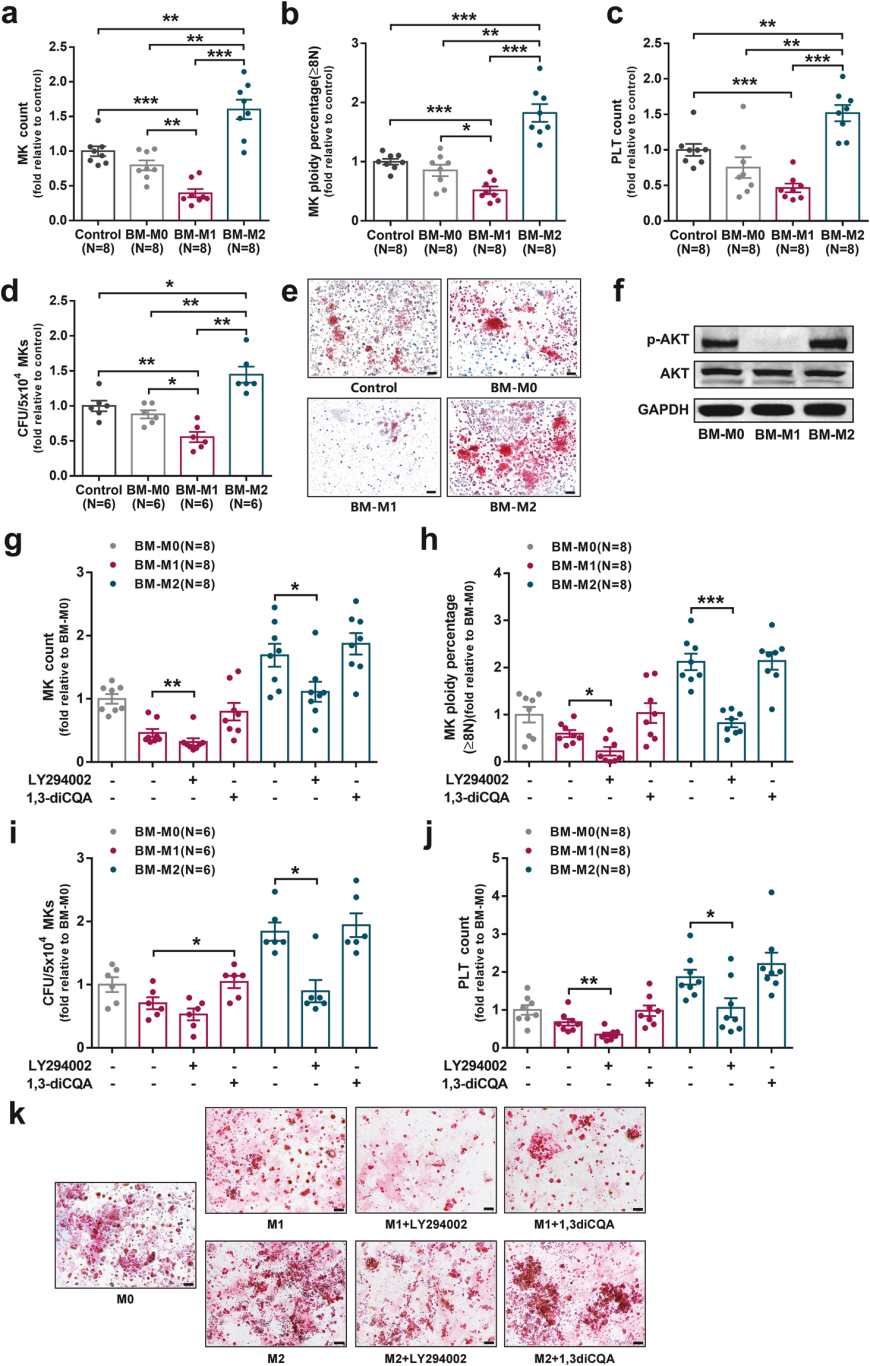
BM-M1 and BM-M2 exerted opposing effects on megakaryopoiesis and platelet production in vitro. The primary BM-M0 from HD were polarized into BM-M1 or BM-M2. Then, direct-contact coculture experiments of the MKs, which were differentiated from BM CD34^+^ cells of HD, with BM-M0, BM-M1 or BM-M2 were performed, respectively. The **a** MK count, **b** MK ploidy distribution, **c** platelet count, **d** CFU-MK count, and **e** representative CFU-MK images (scale bars represent 50 μm) were analyzed after 12 days of coculture. **f** Representative western blots of p-AKT, AKT, and GAPDH in BM-M0, BM-M1, and BM-M2. The MФs were treated with chemical inhibitors LY294002 (10 μM) and activators 1,3-dicaffeoylquinic acid (1,3-diCQA) (10 μM) and the control group was treated with DMSO. The CD34^+^ cells were direct- contact cocultured with the treated MФs. The **g** MK count, **h** MK ploidy distribution, **i** CFU-MK count, **j** platelet count and **k** representative CFU-MK images (scale bars represent 50 μm) were analyzed after 12 days of coculture. Data are presented as the means ± SEM (*P ≤ 0.05, ** *P* ≤ 0.01, *** *P* ≤ 0.001)

Compared with MKs alone culture (control group), direct-contact coculture of BM-M1 with MKs showed significantly decreased MK count (Fig. 4a; 0.40 ± 0.06-fold, *P* = 0.0002), percentage of high-ploidy MKs (Fig. 4b and Supplementary Fig. S2i; 0.52 ± 0.06-fold, *P* = 0.0003), number of CFU-MKs (Fig. 4d,e; 0.56 ± 0.07-fold, *P* = 0.004) and platelet count (Fig. 4c; 0.46 ± 0.06-fold, *P* = 0.0005). Conversely, direct-contact coculture of BM-M2 with MKs showed a significant increase in MK count (Fig. 4a; 1.6 ± 0.14-fold, *P* = 0.003), percentage of high-ploidy MKs (Fig. 4b and Supplementary Fig. S2i; 1.82 ± 0.15-fold, *P* = 0.0002), number of CFU-MKs (Fig. 4d, e; 1.45 ± 0.11-fold, *P* = 0.01) and platelet count (Fig. 4c; 1.52 ± 0.11-fold, *P* = 0.002) compared with the control group. Thus, in direct-contact coculture conditions, BM-M2 showed a greater megakaryopoiesis-supporting capacity compared with BM-M1, as indicated by increases in MK count (Fig. 4a; 1.6 ± 0.14-fold vs. 0.04 ± 0.06-fold, *P* = 0.0002), percentage of high-ploidy MKs (Fig. 4b and Supplementary Fig. S2i; 1.82 ± 0.15-fold vs. 0.52 ± 0.06-fold, *P* = 0.0002), number of CFU-MKs (Fig. 4d, e; 1.45 ± 0.11-fold vs. 0.56 ± 0.07-fold, *P* = 0.002) and platelet count (Fig. 4c; 1.52 ± 0.11-fold vs. 0.46 ± 0.06-fold, *P* = 0.0002).

In indirect-contact coculture conditions, BM-M1 and BM-M2 showed similar tendencies to those in direct-contact coculture conditions. Compared with the control group, the indirect-contact coculture of BM-M1 with MKs showed a decreased megakaryopoiesis-supporting capacity, whereas BM-M2 showed an increased megakaryopoiesis-supporting capacity (Supplementary Fig. S2e–h).

Taken together, these results suggest that M2 MФs support MK maturation and platelet formation, whereas M1 MФs suppress the processes in both direct-contact and indirect-contact conditions. However, the effects of BM-M1 or BM-M2 on megakaryopoiesis were more significant in direct-contact than that in indirect-contact coculture conditions.

### Inhibiting the PI3K-AKT pathway reduced the megakaryopoiesis-supporting ability of M2 MФs

To investigate whether pharmacological inhibition or activation of the PI3K-AKT pathway regulates the effects of MФs on megakaryopoiesis, primary human BM MФs from HD were treated with the PI3K-AKT pathway inhibitor LY294002 or activator 1,3-diCQA and then direct-contact coculture experiments were performed. As shown in Fig. 4f, BM-M2 expressed higher levels of p-AKT than BM-M1. After treated with PI3K-AKT pathway inhibitor, BM-M1 showed an enhanced suppressive effect on megakaryopoiesis as indicated by further decreases in MK count (Fig. 4g; 0.32 ± 0.06-fold vs. 0.46 ± 0.06-fold, *P* = 0.005), percentage of high-ploidy MKs (Fig. 4h; 0.23 ± 0.09-fold vs. 0.60 ± 0.08-fold, *P* = 0.01) and platelet count (Fig. 4j; 0.35 ± 0.05-fold vs. 0.68 ± 0.09-fold, *P* = 0.003) compared to those untreated. Conversely, PI3K-AKT pathway activator ameliorated the suppressive effect of BM-M1 on megakaryopoiesis as indicated by increases in MK count (Fig. 4g; 0.79 ± 0.14-fold vs. 0.46 ± 0.06-fold, *P* = 0.05) and number of CFU-MK (Fig. 4i, k; 1.04 ± 0.10-fold vs. 0.70 ± 0.09-fold, *P* = 0.03). Importantly, PI3K-AKT pathway inhibitor decreased the megakaryopoiesis-supporting ability of BM-M2 as indicated by decreases in MK count (Fig. 4g; 1.11 ± 0.16-fold vs. 1.69 ± 0.18-fold, *P* = 0.04), percentage of high-ploidy MKs (Fig. 4h; 0.82 ± 0.09-fold vs. 2.12 ± 0.17-fold, *P* = 0.0002), number of CFU-MK (Fig. 4i, k; 0.9 ± 0.18-fold vs. 1.84 ± 0.14-fold, *P* = 0.03) and platelet count (Fig. 4j; 1.06 ± 0.25-fold vs. 1.86 ± 0.2-fold, *P* = 0.04) compared to those untreated.

To further confirm the importance of PI3K-AKT pathway in regulating the megakaryopoiesis-supporting ability of M2 MФs, MФs derived from THP1 cells (THP1-M0) were polarized to M1 MФs (THP1-M1) and M2 MФs (THP1-M2). THP1-M2 were treated with the PI3K-AKT pathway inhibitors LY294002 and MK2206. THP1-M2 showed increased levels of p-AKT compared with those in THP1-M1 (Supplementary Fig. S3a). Moreover, the level of p-AKT was decreased in THP1-M2 treated with LY294002 or MK2206. Compared with THP1-M0, THP1-M1 exhibited decreased megakaryopoiesis-supporting ability, as indicated by decreases in MK count (Supplementary Fig. S3b), number of CFU-MK (Supplementary Fig. S3c, e), platelet count (Supplementary Fig. S3d) and percentage of high-ploidy MKs (Supplementary Fig. S3f). By contrast, THP1-M2 showed enhanced megakaryopoiesis-supporting ability compared to THP1-M0 (Supplementary Fig. S3b–f). These results further confirmed that M1 and M2 MФs have opposing effects on megakaryopoiesis that M1 MФs suppress megakaryopoiesis whereas M2 MФs support megakaryopoiesis.

In vitro treatment with PI3K-AKT pathway inhibitor, LY294002 or MK2206 significantly decreased the megakaryopoiesis-supporting ability of THP1-M2 compared with untreated THP1-M2, as shown by decreases in MK count, number of CFU-MK, platelet count, and number of high-ploidy MKs (Supplementary Fig. S3b–f). These findings indicated that inhibiting the PI3K-AKT pathway can functionally impair M2 MФs and reduce their megakaryopoiesis-supporting ability.

### The megakaryopoiesis-supporting ability of MФs was improved by activating the PI3K-AKT pathway

**Fig. 5:**
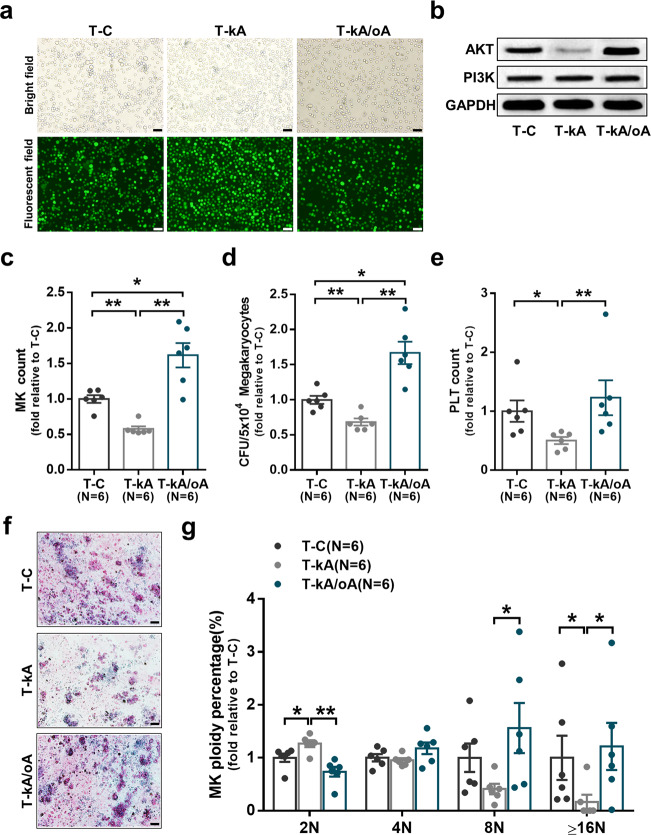
The megakaryopoiesis-supporting ability of macrophages was improved by activating the PI3K-AKT pathway. **a** Representative fluorescence microscopy images of THP1-derived MФs infected with lentivirus (scale bars represent 50 μm). **b** Representative western blots of AKT, PI3K and GAPDH in THP1-derived MФs infected with lentivirus. MK production and maturation, CFU-MK plating efficiencies, and platelet release were analyzed after coculture with the infected MФs. The **c** MK count, **d** CFU-MK count, **e** platelet count, **f** representative CFU-MK images (scale bars represent 50 μm), and **g** MK ploidy distribution were analyzed after 12 days of coculture. Data are presented as the means ± SEM (*P ≤ 0.05, ** *P* ≤ 0.01)

To further investigate whether activating PI3K-AKT pathway improves MФ function by reconstituted expression of Akt1, control lentivirus, Akt1 short hairpin RNA (shRNA) lentivirus, and Akt1 lentivirus were constructed. As shown in Fig. 5a, the lentiviruses in the control group (T-C), Akt1 knockdown group (T-kA), and Akt1 overexpression group (T-kA/oA) effectively infected THP1-derived MФs. The MФ phenotypes in T-C, T-kA, and T-kA/oA groups were analyzed by flow cytometry (Supplementary Fig. S3g). The protein level of AKT was significantly decreased in T-kA group and was reconstituted in T-kA/oA group (Fig. 5b). Compared to T-C group, T-kA group showed an impaired ability to support megakaryopoiesis, as indicated by decreases in MK count (Fig. 5c; 0.54 ± 0.01-fold, *P* = 0.004), number of CFU-MK (Fig. 5d, f; 0.68 ± 0.05-fold, *P* = 0.004), platelet count (Fig. 5e; 0.50 ± 0.06-fold, *P* = 0.01) and percentage of high-ploidy MKs (≥16N) (Fig. 5g; 0.17 ± 0.14-fold, *P* = 0.02). Conversely, Akt1 overexpression enhanced the ability of MФs to support megakaryopoiesis, as shown by the increased MK count (Fig. 5c; 1.65 ± 0.12-fold vs. 0.54 ± 0.01-fold, *P* = 0.002), number of CFU-MK (Fig. 5d, f; 1.67 ± 0.16-fold vs. 0.68 ± 0.05-fold, *P* = 0.002), platelet count (Fig. 5e; 1.23 ± 0.30-fold vs. 0.50 ± 0.06-fold, *P* = 0.004) and percentage of high-ploidy MKs (≥16N) (Fig. 5g; 1.22 ± 0.45-fold vs. 0.17 ± 0.14-fold, *P* = 0.02) compared to Akt1 knockdown. These data suggest that activating the PI3K-AKT pathway can improve MФ function, especially in promoting megakaryopoiesis.

### BM MФ-specific PI3K-knockdown mice demonstrated a dramatic drop in megakaryopoiesis and platelet production

**Fig. 6:**
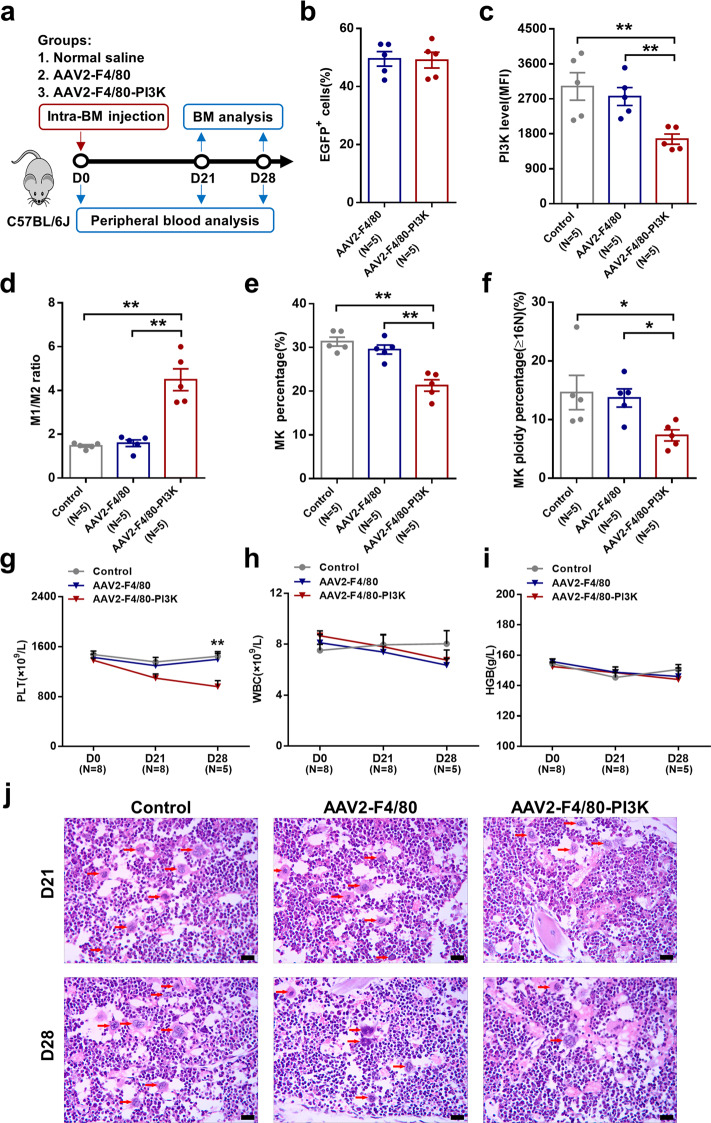
Bone marrow macrophage-specific PI3K-knockdown mice demonstrated a dramatic drop in megakaryopoiesis and platelet production. **a** Schematic diagram of the study design on BM MФ specific PI3K-knockdown mice. **b** Percentage of EGFP^+^ cells in BM CD11b^+^F4/80^+^ cells of AAV2-F4/80 and AAV2-F4/80-PI3K groups. **c** Flow cytometry revealed the intracellular PI3K levels in EGFP^+^ cells. **d** The M1/M2 ratio was calculated to represent the MФ polarization status among control, AAV2-F4/80, and AAV2-F4/80-PI3K groups. The **e** percentage of MKs and **f** percentage of high-ploidy MKs in the BM among control, AAV2-F4/80 and AAV2-F4/80-PI3K groups. Peripheral **g** platelet (PLT), **h** white blood cell (WBC) and **i** hemoglobin (HGB) count among control, AAV2-F4/80 and AAV2-F4/80-PI3K groups. **j** The histopathological analysis of cellularity of BM MKs (×400, red arrows indicate MKs) at day 21 and day 28 after treatment. Data are presented as the means ± SEM (*P ≤ 0.05, ** *P* ≤ 0.01)

To investigate whether PI3K deficiency in BM MФs affects megakaryopoiesis and platelet production in vivo, BM MФ specific PI3K-knockdown mice were constructed using adeno-associated serotype 2 virus (AAV2) viral vectors, which were produced by encoding PI3K shRNA and coexpressing the ZsGreen reporter gene under control of the MФ-specific AAV2-F4/80-PI3K (F4/80) in C57BL/6J mice via intra-BM injection (Fig. 6a). At 4 weeks after injection, flow cytometry showed that the AAV2-F4/80-PI3K were able to efficiently transduce BM MФs in mice as determined by the percentage of EGFP^+^ cells in BM CD11b^+^F4/80^+^ cells (Fig. 6b; 49.1 ± 2.71%). PI3K expression was significantly decreased in the AAV2-F4/80-PI3K-treated mice compared with those treated with the AAV2-F4/80 empty vector (Fig. 6c; 1662 ± 131.2 vs. 3019 ± 354.8, *P* = 0.008). Moreover, AAV2-F4/80-PI3K-treated mice showed a significant increase in M1/M2 ratio in BM (Fig. 6d; 4.49 ± 0.50 vs. 1.59 ± 0.15, *P* = 0.008) compared with those treated with the AAV2-F4/80 empty vector.

We next compared the megakaryopoiesis and platelet production among AAV2-F4/80-PI3K-treated mice, AAV2-F4/80-treated mice, and control groups. The percentage of MKs (Fig. 6e; 21.3 ± 1.31% vs. 29.52 ± 1.05%, *P* = 0.008), percentage of high-ploidy MKs (Fig. 6f; 7.31 ± 0.95% vs. 13.69 ± 1.55%, *P* = 0.016) and peripheral platelet count (Fig. 6g; 960.6 ± 97.19 [×10^9^/L] vs. 1398 ± 96.32 [×10^9^/L], *P* = 0.008) were significantly decreased in AAV2-F4/80-PI3K-treated mice compared with those treated with AAV2-F4/80 empty vector at 4 weeks after treatment. The white blood cells (Fig. 6h) and hemoglobin (Fig. 6i) of AAV2-F4/80-PI3K-treated mice were not significantly different from those treated with AAV2-F4/80 empty vector. Moreover, at 4 weeks after treatment with AAV2 vectors, the cellularity of MKs in BM of AAV2-F4/80-PI3K-treated mice was significantly reduced than that in AAV2-F4/80-treated mice and control group (Fig. 6j). These results demonstrate that M2 MФs support MK maturation and platelet production in vivo, and the process depends on the activation of the PI3K-AKT pathway.

### M2 MФs, which exhibited high TGF-β level and low TNF-α level, enhanced phospho-STAT5 and phospho-ERK activity in cocultured MKs

**Fig. 7:**
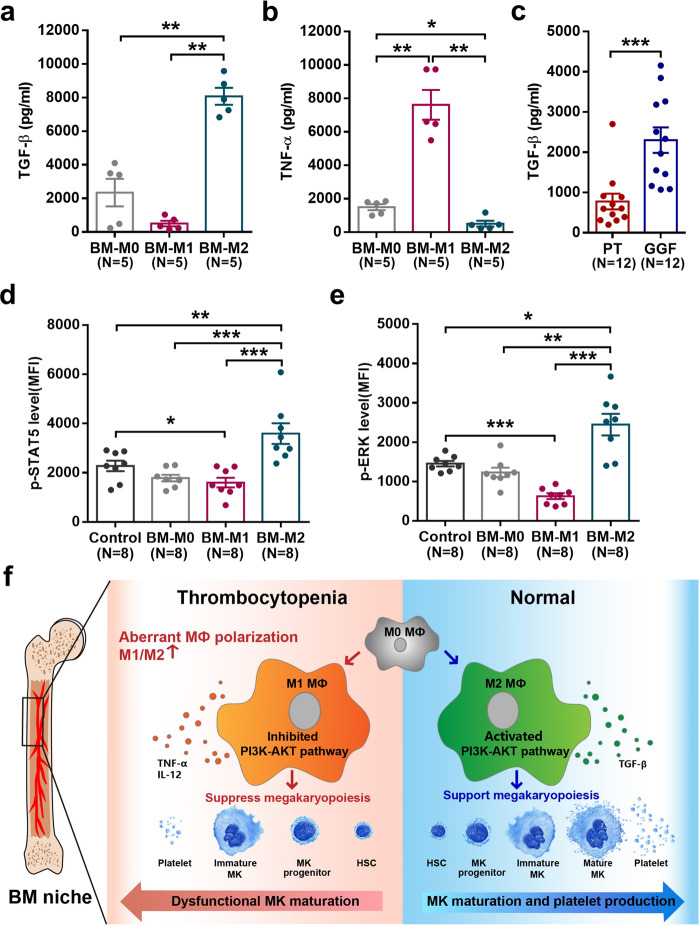
M2 macrophages enhanced phospho-STAT5 and phospho-ERK activity in cocultured megakaryocytes. The ELISA analysis of **a** TGF-β and **b** TNF-α levels in the supernatants of BM MФs. The ELISA analysis of **c** TGF-β levels in the BM plasma of PT or GGF patients. Flow cytometry revealed the intracellular **d** phosphor(p)-STAT5 and **e** p-ERK levels in the MKs after cocultured with the different subtypes of MФs. Data are presented as the means ± SEM (*P ≤ 0.05, ** *P* ≤ 0.01, *** *P* ≤ 0.001). **f** Graphical abstract of the current study. Schematic illustration of the different effects of M1 MФs and M2 MФs on megakaryopoiesis

To study the potential mechanism underlying the enhanced megakaryopoiesis-supporting ability of M2 MФs, the media from the cultivated BM MФs were analyzed using ELISAs. Increased level of TGF-β (Fig. 7a; 8078 ± 503.09 pg/mL vs. 500.2 ± 167.08 pg/mL, *P* = 0.008) was secreted by BM-M2 than by BM-M1. TNF-α level (Fig. 7b; 509 ± 172.04 pg/mL vs. 7611 ± 889.09 pg/mL, *P* = 0.008) was remarkably lower in the supernatants of BM-M2 than in that of BM-M1. Moreover, the level of TGF-β (Fig. 7c; 2300 ± 316.07 pg/mL vs. 777 ± 195 pg/mL, *P* = 0.0001) was significantly higher in BM plasma of GGF patients than that in PT patients. To explore the subsequent changes in maturation-related signaling pathways of the MKs after cocultured with the different subtypes of MФs, the intracellular levels of phosphor(p)-STAT5 and p-ERK were analyzed in MKs. Notably higher levels of p-STAT5 (Fig. 7d; 3590 ± 417.09 vs. 1599 ± 196.02, *P* = 0.0002) and p-ERK (Fig. 7e; 2448 ± 273.09 vs. 628.05 ± 72.86, *P* = 0.0002) were detected in MKs cocultured with BM-M2 than those cocultured with BM-M1. These results suggest that TGF-β released by M2 MФs may facilitate MK maturation through upregulation of the STAT5 and ERK pathway, whereas TNF-α released by M1 MФs suppress MK maturation.

## Discussion

The current study demonstrated that M1 MФs and M2 MФs exert opposing effects on megakaryopoiesis. M1 MФs suppress whereas M2 MФs support MK maturation and platelet release in a PI3K-AKT pathway-dependent manner in humans. Genetic regulation of the PI3K-AKT pathway could modulate the megakaryopoiesis-supporting ability of MФs. Moreover, aberrant BM-M1/M2 MФ polarization characterized by decreased M2 MФs and increased M1 MФs leads to the impaired megakaryopoiesis-supporting ability of BM MФs in PT patients, which could be rescued by PI3K-AKT pathway activators in vitro. Our data indicate that altering PI3K-AKT pathway activity to modulate M1/M2 MФ polarization may be a potential therapeutic approach to enhance megakaryopoiesis and platelet production in patients with thrombocytopenia.

Growing evidence supports that M1/M2 MФ polarization governs the protective and pathogenic roles of MФs in normal and pathological states.^26^ Previous studies have shown that MФs can maintain HSC quiescence and retention in the BM microenvironment, whereas the depletion of MФs causes HSC mobilization in normal states.^11–14^ Recently, we reported that increased M1 and decreased M2 MФ polarization reduced the hematopoiesis-supporting ability of MФs in poor graft function patients, a clinical manifestation of pancytopenia after allo-HSCT, suggesting an imbalance in M1/M2 MФ polarization plays an important role in pathological states.^27^ Anna-Rita Migliaccio et al.^28^ reported that peripheral blood mononuclear cells derived CD14^+^CD163^+^ MФs increase the numbers of CD34^+^ hematopoietic stem and progenitor cells (HSPCs) during coculture, suggesting proliferation and/or survival of the CD34^+^ HSPCs are affected by CD14^+^CD163^+^ MФs. Moreover, Luo et al.^29^ found that M2 MФs promote but M1 MФs inhibit HSC self-renewal, and coculture of human umbilical cord blood CD34^+^ cells with M2 MФs resulted in a significant expansion of CD34^+^ cells and long-term SCID mice-repopulating ability, demonstrating that M1 and M2 MФs play different roles in regulating hematopoiesis. Moreover, M1 MФs were reported to engage in higher rates of phagocytosis of platelets than M2 MФs, suggesting that M1 and M2 MФs may exert distinct regulatory functions in platelets.^30^ However, the precise roles of M1 and M2 MФs or MФ polarization in the regulation of MKs and platelet formation are largely unknown. In the current study, we revealed that M1 MФs suppress megakaryopoiesis and platelet formation in humans whereas M2 MФs support these processes. More importantly, we provided further evidence that M1 and M2 MФs play different roles in regulating megakaryopoiesis in patients with PT, which is characterized by megakaryopoiesis failure after allo-HSCT. Based on our previous work and the current study, we speculate that aberrant BM-M1/M2 MФ polarization, especially increased M1 MФs and decreased M2 MФs in BM, hinders megakaryopoiesis, ultimately leading to failed MK maturation and thrombocytopenia. Therefore, modulating M1/M2 MФ polarization may be a potential therapeutic approach to enhance megakaryopoiesis in patients with thrombocytopenia such as PT after allo-HSCT.

Activation of the PI3K-AKT pathway has been reported to be an essential step toward M2 MФ polarization.^31–33^ Luyendyk et al.^32^ reported that activation or overexpression of PI3K or AKT kinases resulted in reduced M1 MФ polarization. Ruckerl et al.^33^ showed that intact PI3K-AKT signaling is an important factor for driving M2 polarization and proliferation in vitro and in vivo. In agreement with previous reports, upregulation of PI3K-AKT pathway activity was observed in M2 MФs in humans, which leads to increased megakaryopoiesis-supporting ability. In vitro treatment with inhibitors of the PI3K-AKT pathway or Akt1 knockdown disrupted the ability of M2 MФs to support megakaryopoiesis and platelet formation. Moreover, the BM MФ specific PI3K-knockdown mice demonstrated a dramatic drop in megakaryopoiesis and platelet production in vivo. Our data provide further evidence that the PI3K-AKT pathway plays a critical role in regulating the megakaryopoiesis-supporting ability of M2 MФs. Consistently, we observed that activation of the PI3K-AKT pathway improved the impaired ability of MФs from PT patients to support megakaryopoiesis in vitro. Therefore, our data indicate that improving M2 MФ polarization by activating the PI3K-AKT pathway may be a potential therapeutic target for patients with thrombocytopenia.

M2 MФs are even heterogeneous, which can be further subdivided into M2a, M2b, M2c, and M2d subsets.^18,19^ Our data indicated that M2a MФs, which are typically induced by IL-4 and IL-13,^19^ supported MK maturation and platelet release. Besides M1/M2 MФs, other BM-derived MФ subtypes also exert regulatory functions in megakaryopoiesis. For example, Xia et al.^34^ showed that mesenchymal stem cells-reprogrammed BM resident MФs, with arginase 1 positive phenotype and tissue-repair features, improved thrombopoiesis in leukemia-bearing mice. Activation of JAK2/STAT5 and MAPK/ERK pathways play crucial roles in MK maturation.^2,35^ Our preliminary data indicated that TGF-β released by M2 MФs may facilitate megakaryopoiesis through upregulation of the JAK2/STAT5 and MAPK/ERK pathways in MKs. However, we are aware that the precise molecular regulatory mechanism on how MФs mediate megakaryopoiesis needs to be further explored by performing RNA-seq using M1 and M2 MФs directly isolated from BM and tracking phenotypes of both MФs and MKs during coculture in the future.

In summary, the current study demonstrated that M1 MФs and M2 MФs exert opposing effects on megakaryopoiesis and the PI3K-AKT pathway is essential for M2 MФs to support megakaryopoiesis. Defective M2 MФ polarization in BM may be responsible for the occurrence of PT. Although further validation is required, our data may provide new insights into the underlying mechanism and potential therapeutic strategies for patients with megakaryopoiesis failure and thrombocytopenia.

## Materials and methods

### PT patients and their matched controls

A prospective case-control study was conducted to evaluate the polarization of BM MФs from PT patients and their matched patients with GGF. Transplant recipients were identified from patients who received an allotransplant between March 1, 2018 and August 10, 2020 at the Peking University Institute of Hematology. A total of 17 patients who developed PT were eligible. For each PT patient enrolled, transplant recipients with GGF (*N* = 34) were selected from the same cohort after matching for age, pretransplant disease state and posttransplant interval (risk-set sampling). None of the clinical characteristics, such as the transplanted CD34^+^ cell dose, history of graft versus host disease (GvHD), or cytomegalovirus (CMV) infection, showed significant differences between PT patients and GGF patients (Table 1). Donor selection, HLA typing, graft harvesting, conditioning therapy, and GvHD prophylaxis were performed as previously reported.^36–39^ BM samples from HD (*N* = 30) were normal controls.

**Table 1:** Characteristics of allo-HSCT patients with PT and GGF

Characteristics	PT^a^ (*N* = 17)	GGF^a^ (*N* = 34)	*P-*value**
BM evaluated time (post-HSCT days)	64 (58–175)	63 (58–178)	0.67
Blood cell count			
Median WBC (×10^9^/L) (range)	3.20 (1.37–6.57)	4.29 (1.79–6.50)	0.08
Median ANC (×10^9^/L) (range)	2.57 (0.91–3.80)	3.00 (0.98-5.20)	0.31
Median Hb (g/L) (range)	83 (73–126)	108 (80–153)	<0.0001
Median PLT (×10^9^/L) (range)	22 (13–38)	130 (82–239)	<0.0001
Age at HSCT (years, median, range)	35 (18–60)	35 (18–64)	0.90
Gender (male/female)	9/8	20/14	0.69
Underlying disease			1.00
AML	9	18	
ALL	5	10	
MDS	3	6	
Status at HSCT			0.74
Standard-risk	15	31	
High-risk	2	3	
Source of stem cell			1.00
BM and PB	17	34	
Transplanted total nucleated cell dose (×10^8^/kg, median, range)	8.40 (7.05–9.84)	8.88 (3.98–9.98)	0.56
Transplanted CD34^+^ cell dose (×10^6^/kg, median, range)	2.05 (1.06–5.89)	2.11 (1.02–5.85)	0.89
Donor match			1.00
HLA-identical sibling donor	0	0	
HLA-partially matched related donor	17	34	
Sex mismatch			0.94
Female to male	4	6	
Female to female	1	3	
Male to female	7	14	
Male to male	5	11	
ABO mismatch			0.27
No	13	18	
Minor	2	8	
Major	2	8	
Pre-HSCT cycles of chemotherapy	3 (0–6)	3 (0–7)	0.49
Conditioning			1.00
BU/CY	0	0	
BU/CY + ATG	17	34	
History of CMV reactivation	11	20	0.69
Onset of CMV reactivation (days, median, range)	28 (8–72)	24 (7–56)	0.21
CMV reactivation treated with ganciclovir	10	15	0.32
History of GvHD	7	15	0.84
Onset of aGvHD (days, median, range)	17 (7–60)	14 (5–62)	0.57

**The continuous variables were compared using the Mann–Whitney *U*-test, and the differences in frequency between the two groups were compared using the chi-square test. The criterion for statistical significance was *P* < 0.05

^a^Group matching criteria included age at HSCT (±1years), pre-HSCT cycles of chemotherapy (±1cycle), disease status at HSCT, and BM microenvironment evaluated time after HSCT (±5 days). For each case, two GGF control was randomly selected from the same cohort at which the PT occurred (risk-set sampling)

This study was approved by the Ethics Committee of Peking University People’s Hospital, and written informed consent was obtained from all subjects in compliance with the Declaration of Helsinki.

### Definition of PT and GGF

As previously reported,^10^ PT was defined as engraftment of all peripheral blood cell lines (absolute neutrophil cells >0.5 × 10^9^/L and hemoglobin >70 g/L without transfusion support) other than a platelet count <20 × 10^9^/L or dependence on platelet transfusions for more than 60 days following allo-HSCT in the presence of complete donor chimerism. GGF was defined as persistent successful engraftment beyond 28 days after HSCT.^7–10,27,40^ Patients with evidence of poor graft function, severe GvHD or hematologic relapse following allo-HSCT were excluded.

### Analysis of monocyte and MФ subsets

For analysis of classical, intermediate and non-classical monocytes, cells were stained with a phycoerythrin (PE)-conjugated anti-CD14 monoclonal antibody (mAb) and an APC-Cy7-conjugated anti-CD16 mAb (BioLegend, San Diego, CA, USA). The analysis was performed according to a standardized gating strategy.^27,41^ Briefly, the total monocytes were gated based on forward scatter (FSC)/side scatter (SSC) properties. The classical monocytes (CD14^++^CD16^−^), the intermediate monocytes (CD14^++^CD16^+^), and the non-classical monocytes (CD14^+^CD16^++^) were gated based on the expression of CD14 and CD16. For more definitive identification of primary pre-cultivated BM MФ subsets,^42^ FITC-conjugated anti-CD68 and BV421-conjugated anti-CCR2 (BioLegend) mAbs were used for the identification of M1 MФs (CD68^+^CCR2^+^), whereas PerCP/Cy5.5-conjugated anti-CX3CR1 and PE/Cy7-conjugated anti-CD163 (BioLegend) mAbs were used for the identification of M2 MФs (CD163^+^CX3CR1^+^). The relative frequencies of these monocyte subsets were expressed as percentages of the total gated monocytes. To determine the intracellular cytokines in MФs, PE/Cy7-conjugated anti-TNF-ɑ, PE-conjugated anti-IL-12, and BV421-conjugated anti-TGF-β (BioLegend) mAbs were used and evaluated by LSRFortessa (Becton Dickinson, San Jose, CA, USA) and expressed as the mean fluorescence intensity (MFI) (mean ± SEM).

### Cultivation and functional analyses of human primary BM MФs

Primary human MФs from HD or patients were generated as described previously.^27^ BM mononuclear cells (BMMNCs) were isolated by density gradient centrifugation using a lymphocyte separation medium (GE Healthcare, Milwaukee, WI, USA). Briefly, monocytes isolated from BMMNCs of patients with PT, GGF or HD were differentiated into the cultivated PT MФs, GGF MФs or HD MФs for 7 days in RPMI 1640 medium supplemented with 10% heat-inactivated fetal bovine serum (FBS), 1% penicillin/streptomycin, and 100 ng/mL macrophage colony-stimulating factor (PeproTech, Rocky Hill, NJ, USA). The resulting primary HD MФs were then polarized into M1 (BM-M1) or M2 (BM-M2) MФs by culturing for 24 hours in RPMI 1640 medium supplemented with either 100 ng/mL LPS and 20 ng/mL IFN-γ or 20 ng/mL IL-4 and 20 ng/mL IL-13, respectively. The cultivated BM MФs were evaluated with phagocytosis and migration assays as previously described.^27^ The supernatant of MФs and the BM plasma of patients with PT or GGF were harvested. The levels of TNF-α and TGF-β were measured using ELISA kit (Abcam, Cambridge, UK) following the manufacturer’s instructions.

### Coculture of BM CD34^+^ cells with MФs

BM CD34^+^ cells were isolated from BMMNCs of HD using a CD34 MicroBead kit (Miltenyi Biotec, Bergisch Gladbach, Germany) as previously described.^7,8,27,40,43^ The treated MФs were carefully washed three times with 1 mL 1 × PBS to remove exogenous cytokines and chemical reagents. Subsequently, CD34^+^ cells were added and direct-contact cocultured with the treated MФs for another 7 days in StemSpanTM SFEM (Stem Cell Technologies, Vancouver, BC, Canada) containing 100 ng/mL SCF, 100 ng/mL TPO, and 10 ng/mL IL-3 (PeproTech) to promote MK differentiation.^7,8,43,44^ Indirect-contact coculture assay was conducted simultaneously where CD34^+^ cells were plated on the upper chamber of a Transwell (Corning Incorporated, NY, USA) suspended above the MФs. Appropriate controls of CD34^+^ cells alone and unstimulated MФs cultures were included in coculture experiments. The CFU-MKs were analyzed by a commercially available kit (MegaCult-C; Stem Cell Technologies). A total of 5 × 10^4^ CD34^+^ cells were plated in each chamber slide for 12 days, and MK colonies were defined as groups of 3 or more glycoprotein IIb/IIIa-positive cells. Quantification of the MK count, MK polyploidy distribution, and platelet production in the coculture system was performed as previously described.^7,8,43,44^ To measure intracellular protein levels in MKs after 12 days of coculture, the MKs were identified by labeling with CD41a and then fixed, permeabilized, and incubated with p-STAT5 and p-ERK (Cell Signaling Technology, Danvers, MA, USA). The intracellular protein levels were evaluated by LSRFortessa software (Becton Dickinson) and expressed as the MFI (mean ± SEM).

### Immunofluorescence staining and image analysis

As previously described,^27^ the immunofluorescence staining of the BMBs were performed with the mouse anti-human CD68 (Abcam, MA, USA) and rabbit anti-human CD163 (Abcam) antibodies. 4′,6-Diamidino-2-phenylindole (DAPI) was applied to stain the nuclei, and the slides were analyzed under a Leica TCS SP8 microscope (Leica Microsystems, Wetzlar, Germany). Positive cells per HPF were quantified in a semiautomated way using ImageJ software.

### RNA sequencing and data analysis

The cultivated BM MФs samples, including the primary MФs from PT and GGF patients, and the polarized BM-M1 and BM-M2 from HD, were analyzed using RNA-seq. Briefly speaking, next-generation RNA-seq libraries were constructed with qualified RNA samples using the NEBNext® Ultra™ RNA Library Prep Kit for Illumina. The eligible libraries were sequenced on an Illumina HiSeq XTen platform (150bp paired-end reads), yielding ~6G raw data per sample. Low-quality reads and adapter sequences were removed and the remained clean reads were quantified against an Ensembl catalog (GRCh38) at the transcript level by Salmon software and aggregated at the gene level using the R package “tximport”.^45^ Finally, the transcripts per kilobase million (TPM) and count value were used for the following analysis. Differentially expressed genes (DEGs) were calculated with R package “DESeq2” and “IHW”. With the DEGs, GO enrichment analysis and their hierarchy relation analysis were conducted with the R package “topGO”. KEGG pathway analyses were performed with the R package “clusterProfiler”. To determine the MФ phenotype scores from the samples of patients, a deconvolution approach was performed using an R-based version of CIBERSORT with gene list “immunoState”.^46^ The MФ phenotype scores include M1 and M2 phenotype, which named after M1 and M2 score, respectively. To some extent, the score integrated a more comprehensive phenotype of MФs compared with flow cytometry data.

### Western blot analysis

Total proteins obtained from cell lysates were subjected to SDS-PAGE and transferred onto a polyvinylidene difluoride membrane (Bio-Rad Laboratories, Hercules, CA, USA). For experiments involving signaling pathways, antibodies against PI3K p110α, AKT, and p-AKT (Ser473) (Cell Signaling Technology) were used. Membranes were probed with primary antibodies at 4 °C overnight, followed by incubation of anti-rabbit or anti-mouse secondary antibodies. The membranes were developed with ECL reagents (Millipore, Bedford, MA) before protein bands were observed on X-ray films. To control for total protein loading, membranes were stripped of the primary antibodies and reprobed with anti-GAPDH antibody (Sigma Chemical Co., St. Louis, MO, USA). Quantification of the band intensities was performed using GeneTools software (Syngene).

### Cultivation and polarization of THP1 MФs

THP1, a human promonocytic cell line derived from a patient with an acute monocytic leukemia, was obtained from the American Type Culture Collection (Manassas, VA, USA) and cultured in RPMI 1640 medium supplemented with 10% FBS and 1% penicillin/streptomycin. Cells were differentiated to THP1-M0 by first incubating them with 320 nM phorbol 12-myristate 13-acetate (PMA; Sigma) for 24 h. To generate THP1-M1, THP1-M0 were treated with M1 cytokines (100 ng/mL LPS and 20 ng/mL IFN-γ) for an additional 24 h. To generate THP1- M2, THP1-M0 were exposed to the M2 cytokines (IL-4 and IL-13, both 20 ng/mL) for another 24 h.

### Reagent treatment and transfection of MФs in vitro

MФs were treated with chemical inhibitors LY294002 (10 μM) or MK2206 (10 μM) or activators 1,3-dicaffeoylquinic acid (1,3-diCQA) (10 μM) or YS-49 (10 μM) (MedChemExpress, Monmouth Junction, NJ) of the PI3K-AKT pathway for 24 hours.^47^ The control group was treated with DMSO. Lentivirus expressing a shRNA targeting Akt1 and lentivirus selectively expressing Akt1, both of which were synthesized by Shanghai GeneChem Co., Ltd. (Shanghai, China), were added to THP1-derived MФs to knockdown or overexpress Akt1, respectively. For the control, a lentiviral vector that expresses GFP alone was used. MФs were cultured for 72 h following infection and selected in puromycin (2 μg/mL). Effective knockdown and overexpression were evaluated by fluorescence microscopy and western blot analysis.

### Establishment of a BM MФ specific PI3K-knockdown murine model

To establish a BM MФ specific PI3K-knockdown murine model, we used the AAV2 viral vectors to deliver MФ-specific AAV2-F4/80-PI3K (F4/80) promoter to control PI3K shRNA vectors to BM MФs in C57BL/6J mice via intra-BM injection. Recombinant AAV2 was generated using pAAV-CR and pHelper in HEK293 cells as described previously.^48,49^ AAV2 viral vectors were produced encoding PI3K shRNA and coexpressing the ZsGreen reporter gene under the control of the MФ-specific AAV2-F4/80-PI3K promoter.^50,51^ Empty vector under the control of the MФ-specific AAV2-F4/80 promoter was constructed at the same time. AAV2-F4/80-PI3K viral titer of 1.2 × 10^13^ vector genomes (vg)/mL, AAV2-F4/80 viral titer of 1.2 × 10^13^ vg/mL were used in the study.

For local delivery, 60 μL of AAV2-F4/80-PI3K or AAV2-F4/80 were intraosseously injected into BM of 6-week-old male C57BL/6J mice (*n* = 8). The control group was injected with normal saline. To analyze the recovery kinetics in peripheral blood, retro-orbital blood was collected from mice before injection and at 3 and 4 weeks after injection and analyzed using an automated hematology analyzer (Sysmex Corporation, Japan).

The GFP-positive cells in BM were measured by flow cytometry. To evaluate the MФ subsets in the BM of mice, femurs and tibias of each mouse were collected to make single-cell suspensions, and then stained with anti-CD11b-APC-Cy7, anti-F4/80-BV421, anti-CD86-PE, and anti-CD163-APC (BioLegend) and analyzed by flow cytometry. BM MK polyploidy distribution were evaluated after labeling with CD41a and then fixed and incubated with PI staining buffer.

For histopathological analysis of BM, three mice per group were sacrificed at 3 weeks and five mice per group were sacrificed at 4 weeks after injection, and the femurs were harvested and fixed in 4% paraformaldehyde for hematoxylin and eosin (H&E) staining.

### Statistical analyses

Statistical analyses were performed using one-way analysis of variance (ANOVA) for comparisons among groups. Subject variables were compared using a chi-squared test for categorical variables and a Mann–Whitney *U*-test for continuous variables. Wilcoxon’s test for paired data was used to identify the drug effects. Analyses were performed with SPSS version 25.0 (SPSS Inc, IBM, Chicago, IL, USA) and GraphPad Prism 6.0 (GraphPad Software, La Jolla, CA, USA), and *P*-values < 0.05 were considered significant. Data are presented as the means ± SEM.

## Supplementary information

XJH_Supplementary materials (SIGTRANS-02600R)

## Data Availability

The data that support the findings of this study are available from the corresponding author upon reasonable request.
