# Correction: M2 macrophages, but not M1 macrophages, support megakaryopoiesis by upregulating PI3K-AKT pathway activity

**DOI:** 10.1038/s41392-024-01965-3

**Published:** 2024-09-12

**Authors:** Hong-Yan Zhao, Yuan-Yuan Zhang, Tong Xing, Shu-Qian Tang, Qi Wen, Zhong-Shi Lyu, Meng Lv, Yu Wang, Lan-Ping Xu, Xiao-Hui Zhang, Yuan Kong, Xiao-Jun Huang

**Affiliations:** 1 Peking University People’s Hospital, Peking University Institute of Hematology, National Clinical Research Center for Hematologic Disease, Beijing Key Laboratory of Hematopoietic Stem Cell Transplantation, Collaborative Innovation Center of Hematology, Peking University, Beijing, China; 2 Peking-Tsinghua Center for Life Sciences, Academy for Advanced Interdisciplinary Studies, Peking University, Beijing, China

Correction to: *Signal Transduction and Targeted Therapy* 10.1038/s41392-021-00627-y, published online 18 June 2021

In the process of collating the raw data, the authors noticed three images in Fig. 4 were used incorrectly. They made an inadvertent mistake by arranging the figure of M2 Group into M2+LY294002 Group in Fig. 4k during the revision process.
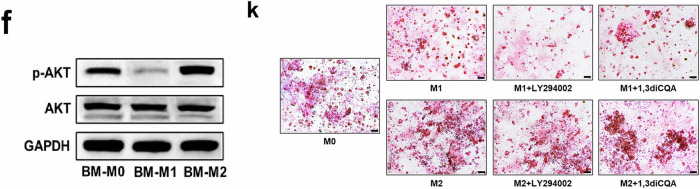


The correct Fig. 4 is provided as follows:
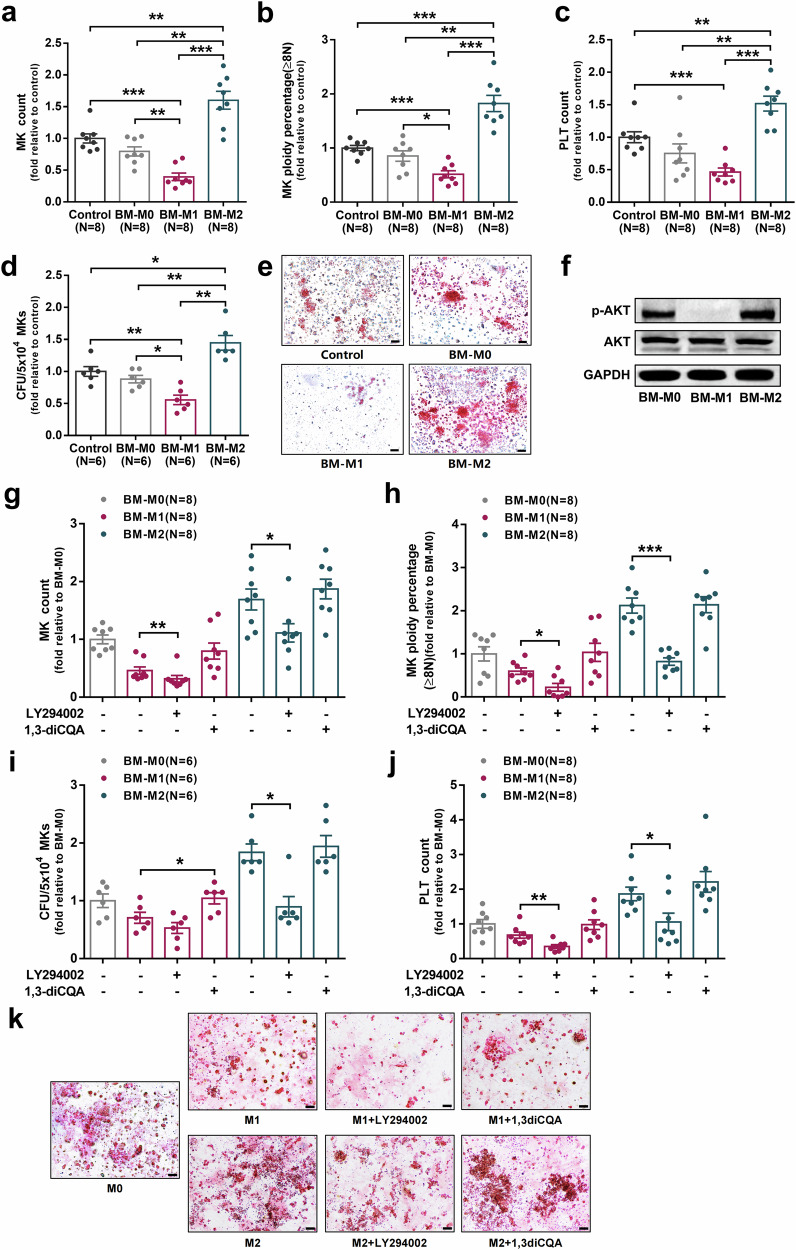


Fig. 4 BM-M1 and BM-M2 exerted opposing effects on megakaryopoiesis and platelet production in vitro. (f) Representative western blots of p-AKT, AKT and GAPDH in BM-M0, BM-M1 and BM-M2. (k) Representative CFU-MK images (scale bars represent 50 μm) were analyzed after 12 days of coculture.

In addition, the bands of Akt and GAPDH in Fig. 5b were vertically flipped and inserted as p-Akt and GAPDH in Fig. 4f by mistake.

The correction did not affect any of the results or discussion as present in the original publication. The authors apologize for this inadvertent mistake.

The original article has been corrected.

