# Supplementary material for: M2 macrophages, but not M1 macrophages, support megakaryopoiesis by upregulating PI3K-AKT pathway activity
Source: Signal Transduct Target Ther. 2021 Jun 18;6:234. doi: 10.1038/s41392-021-00627-y (PMC8211642; doi:10.1038/s41392-021-00627-y)
Supplement: Supplementary file 1 — XJH_Supplementary materials (SIGTRANS-02600R) [file 41392_2021_627_MOESM1_ESM.docx]

**Supplementary Materials for**

**M2 macrophages, but not M1 macrophages, support megakaryopoiesis by upregulating PI3K-AKT pathway activity**

**Authors:** Hong-Yan Zhao^1^, Yuan-Yuan Zhang^1^, Tong Xing^1,2^, Shu-Qian Tang^1^, Qi Wen^1^, Zhong-Shi Lyu^1,2^, Meng Lv^1^, Yu Wang^1^, Lan-Ping Xu^1^, Xiao-Hui Zhang^1^, Yuan Kong^1^*****, Xiao-Jun Huang^1,2^*****

**^1^**Peking University People’s Hospital, Peking University Institute of Hematology, National Clinical Research Center for Hematologic Disease, Beijing Key Laboratory of Hematopoietic Stem Cell Transplantation, Collaborative Innovation Center of Hematology, Peking University, Beijing, China;

**^2^**Peking-Tsinghua Center for Life Sciences, Academy for Advanced Interdisciplinary Studies, Peking University, Beijing, China.

*****Xiao-Jun Huang and Yuan Kong are co-corresponding authors.

Correspondence to: Xiao-Jun Huang**:** huangxiaojun@bjmu.edu.cn

Yuan Kong**:** [successky@163.](mailto:successky@163.)com

**This PDF file includes:**

Figures S1-S3


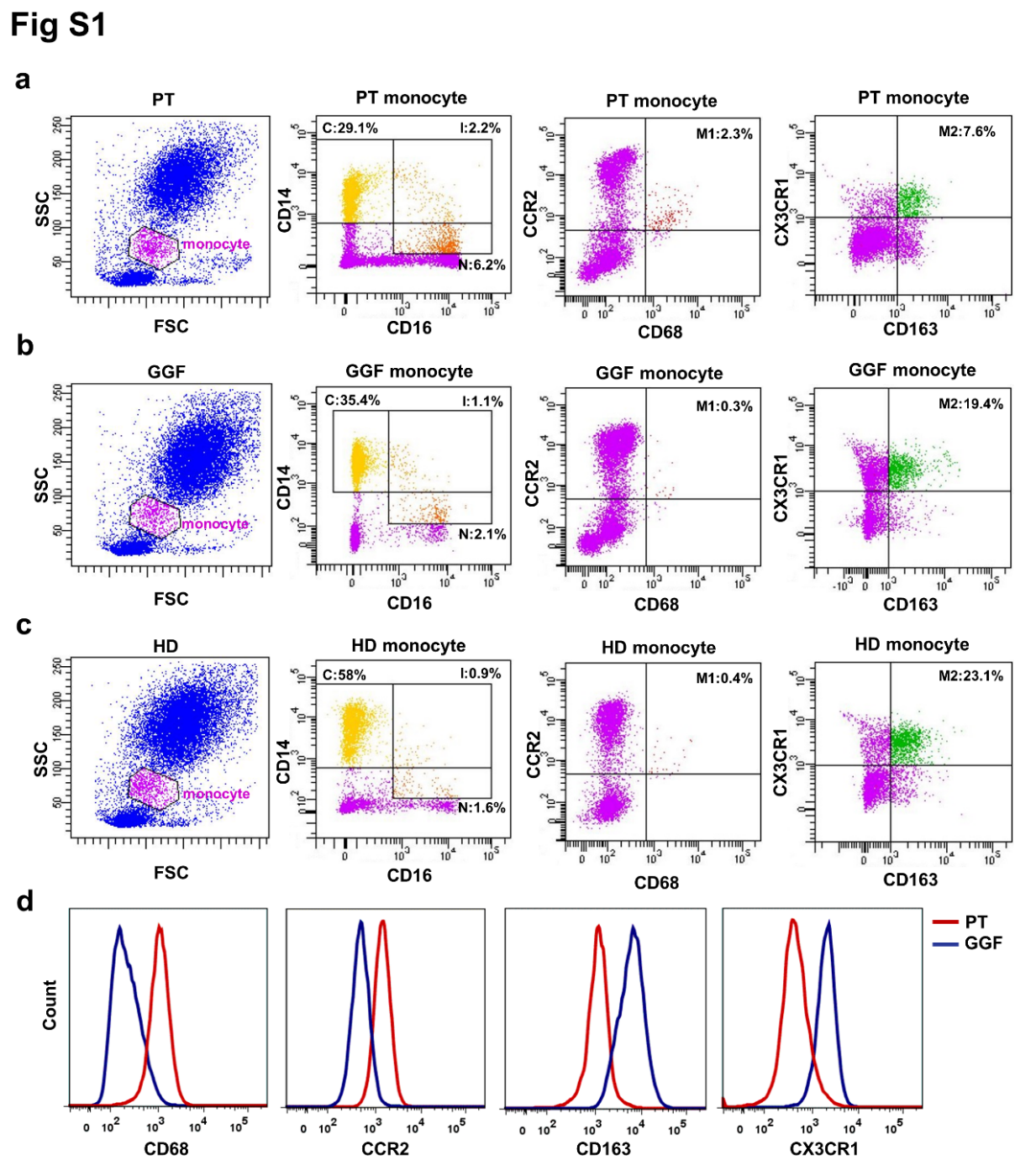


**Fig.S1: Altered distribution of monocyte-MФ subsets in the BM of PT patients.** Representative scatter plots of classical CD14^++^CD16^-^ monocytes(C), intermediate CD14^++^CD16^+^ monocytes(I), non-classical CD14^+^CD16^+^ monocytes(N), BM M1(CD68^+^CCR2^+^) and M2(CX3CR1^+^CD163^+^) cells among **(a)**PT patients, **(b)**GGF patients and **(c)**HD. **(d)**The phenotypes of MФs cultured from monocytes isolated from the BM(cultivated BM MФs) of PT patients and GGF patients were analyzed by flow cytometry.


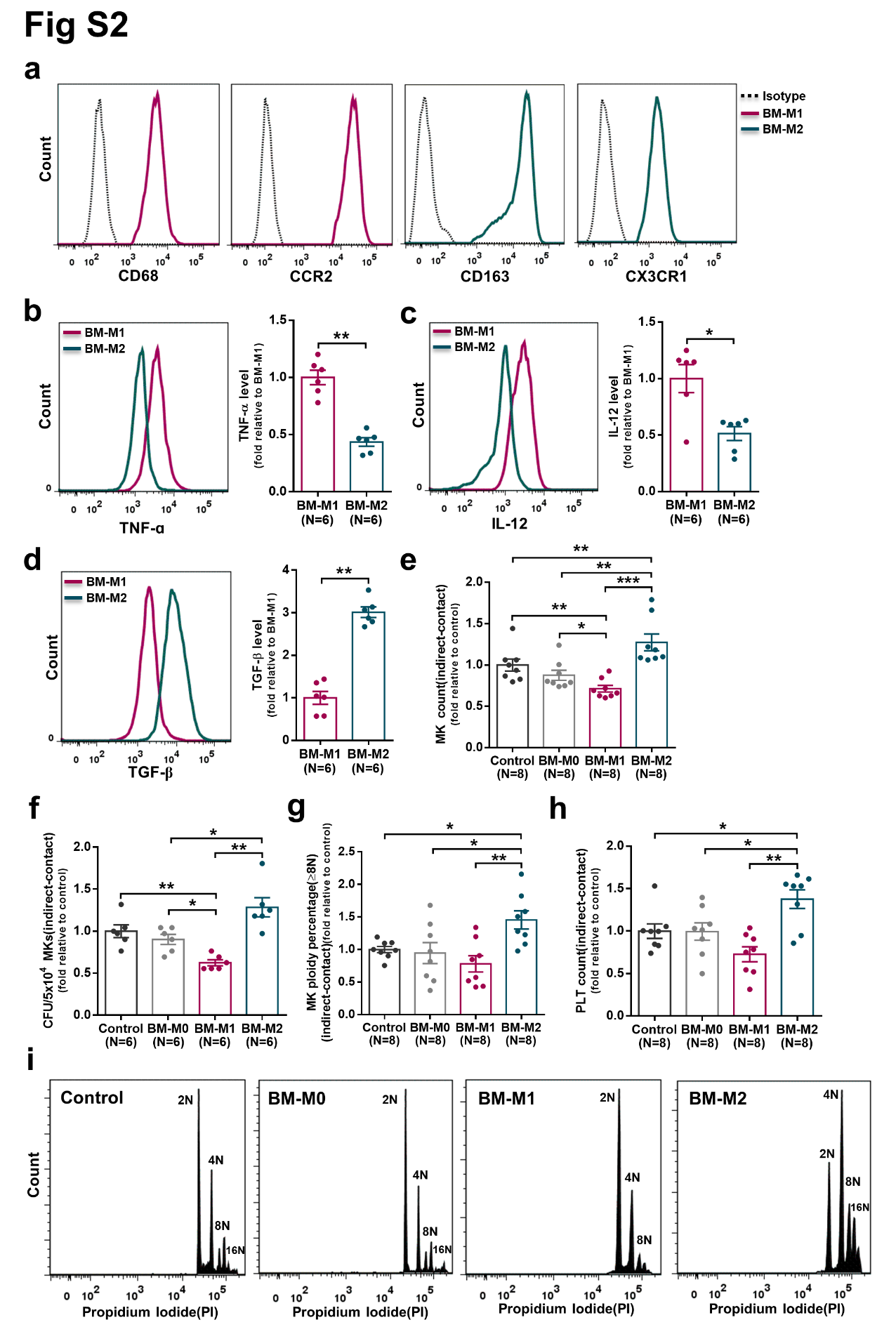


**Fig.S2: BM-M1 and BM-M2 exerted opposing effects on megakaryopoiesis and platelet production in vitro. (a)** The typical M1 and M2 macrophage(MФ) phenotypes were confirmed by demonstrating the positive expression of CD68, CCR2 or CD163 and CX3CR1 by flow cytometry(full line). Aliquots of isotype-identical antibodies served as negative controls(dotted line). Intracellular levles of **(b)** TNF-ɑ, **(c)** IL-12 and **(d)** TGF-ꞵ were analyzed in BM-M1 and BM-M2 MФs. The primary BM-M0 from HD were polarized into BM-M1 or BM-M2. Then, indirect-contact coculture experiments of the MKs, which were differentiated from BM CD34^+^ cells of HD, with BM-M0, BM-M1 or BM-M2 were performed, respectively. The **(e)** MK count, **(f)** CFU-MK count, **(g)** MK ploidy distribution, and **(h)**platelet count were analyzed after 12 days of coculture. **(i)** Representative MK ploidy distribution images of MKs after direct-contact cocultured with the MФs. Data are presented as the means ± SEM (**P*≤ 0.05, ** *P*≤ 0.01, *** *P*≤ 0.001).

**
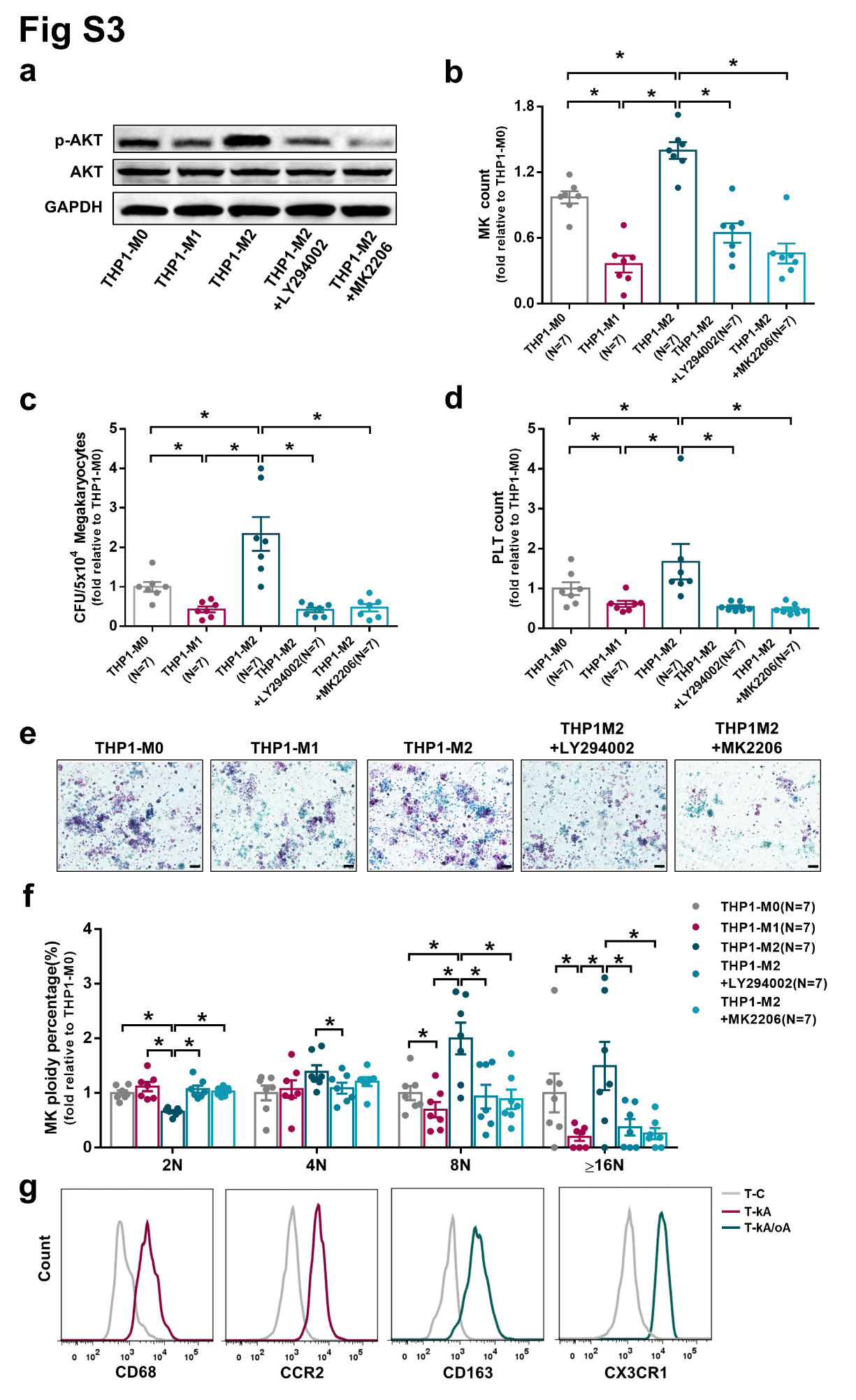
**

**Fig.S3: Inhibiting the PI3K-AKT pathway reduced the megakaryopoiesis-supporting ability of M2 Macrophages. (a)** Representative western blots of p-AKT, AKT and GAPDH expression in THP1-derived MФs cultured in the presence or absence of PI3K-AKT pathway inhibitors(LY294002, 10 μM; MK2206, 10 μM). MK production and maturation, colony-forming unit MK(CFU-MK) plating efficiencies, and platelet release were analyzed after coculture with the treated MФ subsets. The **(b)** MK count, **(c)** CFU-MK count, **(d)** platelet count, **(e)** representative CFU-MK images(scale bars represent 50 μm) and **(f)** MK ploidy distribution were analyzed after 12 days of coculture. **(g)** The phenotypes of infected THP1-derived MФs among control group(T-c), Akt1 knockdown group(T-kA) and Akt1 overexpression group(T-kA/oA) were analyzed by flow cytometry. Data are presented as the means ± SEM (**P*≤ 0.05)
